# Urine Proteomics for Detection of Potential Biomarkers for End-Stage Renal Disease

**DOI:** 10.3390/ijms26125429

**Published:** 2025-06-06

**Authors:** Nathalia R. Silva, Bianca U. Picolo, Letícia C. M. de Sousa, Marta S. dos Santos, Richard C. Polveiro, Hebréia O. Almeida-Souza, Mário M. Martins, Luiz R. Goulart Filho, Luciana S. da Silva

**Affiliations:** 1 Faculty of Medicine, Federal University of Uberlândia, Uberlândia 38402-293, Brazil; nathaliarabello@ufu.br (N.R.S.);; 2 Nanobiotechnology Laboratory Prof. Luiz Ricardo Goulart Filho, Institute of Biotechnology, Federal University of Uberlândia, Uberlândia 38405-319, Brazil; 3 Faculty of Veterinary Medicine, Federal University of Uberlândia, Uberlândia 38410-337, Brazil

**Keywords:** biomarkers, chronic kidney disease, disease progression, nephrology, proteomics

## Abstract

The increasing number of individuals with chronic kidney disease (CKD), mainly due to lifestyle changes—such as increased consumption of processed foods, physical inactivity, obesity, and smoking habits—and population aging, highlights the need to identify new biomarkers to facilitate monitoring of CKD progression and, consequently, predict end-stage renal disease (ESRD). This study aimed to analyze the proteomic profile of urine samples from healthy individuals and those with ESRD to identify potential biomarkers for this advanced stage of CKD. Urine samples were collected from 20 participants, comprising 10 healthy individuals and 10 patients with ESRD, and analyzed via liquid chromatography coupled with a tandem mass spectrometer. Bioinformatics analyses, including gene ontology and protein interaction, were subsequently conducted. A total of 416 proteins were identified in the proteomic profiles of the groups, and 19 proteins showed statistically significant differences between them. Of these, five proteins—hemopexin, beta-2-microglobulin, retinol-binding protein 4, transthyretin, and factor D—emerged as potential biomarkers for ESRD. The proteins identified were able to characterize and differentiate the urinary proteomic profiles of the two groups. The five selected proteins represent promising candidates for ESRD biomarkers.

## 1. Introduction

Chronic kidney disease (CKD) is characterized by a progressive loss of renal function associated with the consequent reduction of blood filtration capacity and maintenance of homeostasis [1,2]. According to Kidney Disease: Improving Global Outcomes (KDIGO), CKD is classified into stages based on the glomerular filtration rate (GFR): stage 1 (≥90 mL/min/1.73 m^2^), stage 2 (60–89.9 mL/min/1.73 m^2^), stage 3a (45–59.9 mL/min/1.73 m^2^), stage 3b (30–44.9 mL/min/1.73 m^2^), stage 4 (15–29.9 mL/min/1.73 m^2^), and stage 5 (<15 mL/min/1.73 m^2^) [2]. CKD can be determined by a GFR less than 60 mL/min/1.73 m^2^, which is associated with at least one marker of kidney damage—such as albuminuria—for a period of three months or more, regardless of cause [2].

CKD represents a significant public health challenge, affecting an estimated 843.6 million individuals worldwide [3]. It is the third fastest-growing cause of death globally and the only non-communicable disease (NCD) to demonstrate a sustained increase in age-adjusted mortality rates [4]. CKD is associated with high morbidity and mortality, as well as substantial socioeconomic impacts globally [1]. CKD management has a significant impact on healthcare budgets, with costs increasing as the disease progresses. In England, CKD accounted for GBP 1.45 billion in National Health Service expenses during 2009–2010, while United States Medicare expenditures for CKD and ESKD surpassed USD 114 billion in 2016 [5]. The global median annual cost of maintaining hemodialysis in 2021 was estimated at USD 19,380 per patient, with a range of USD 11,818 to USD 38,005 [6].

Early diagnosis of CKD is essential to reduce the morbidity and mortality of patients through the adoption of measures capable of reducing the progression of CKD to the terminal stage [7]. However, in the initial stages of this disease, few signs and/or symptoms are perceived in the individual, which makes early detection difficult [8]. Added to this is the fact that the parameters currently used are not considered sensitive for the detection of this disease in the initial stages [7]. Creatinine is the most used marker in clinical practice due to its availability and low cost. However, it is not considered a sensitive parameter in the early stages of the disease, which may contribute to early underdiagnosis of CKD [9]. This measurement may also be influenced by several factors, such as the amount of muscle mass, renal tubule secretion, a high-protein diet, and physical activity, among others [10].

Given the above, it is necessary to identify new biomarkers for the diagnosis and monitoring of the progression of CKD [11]. The omics approach is considered a means for the discovery of these biomarkers. Omics technologies involve the comprehensive molecular analysis of cells, tissues, organs, or entire organisms [12]. These approaches allow high-throughput exploration of various biological layers, including the genome, epigenome, proteome, transcriptome, and metabolome [13]. Among the techniques used, the proteomic analysis of urine samples stands out since the collection of this biological material is simple and non-invasive [14]. In addition, urine is considered an important source of biomarkers for various diseases due to specific changes in the proteome [14].

It is known that the proteomic analysis of urine samples has potential for the diagnosis and/or monitoring of the progression of diabetic kidney disease (DRD) [15], primary membranous nephropathy [16], and cardiovascular disease associated with CKD [17], among others.

In the current literature, few studies addressing the topic of urinary proteomics and chronic kidney disease were found. A study published in 2019 found urinary proteomic biomarkers that were able to predict cardiovascular outcomes in patients with CKD in the early stages [17]. In addition, a systematic review showed that proteins capable of estimating the progression of kidney disease were found in patients with specific health conditions, such as nephrotic syndrome, IgA nephropathy, and systemic lupus erythematosus, among others [18]. Therefore, the present study is important because it unearths new information regarding urinary proteomics and end-stage renal disease (ESRD).

Thus, the aim of this study was to analyze the proteomic profile of urine samples from healthy individuals and those with ESRD to identify potential biomarkers for this advanced stage of CKD.

## 2. Results

### 2.1. Characterization of the Participants

Table 1 shows the sociodemographic, behavioral, and biochemical variables of the sample studied. It is noteworthy that in the hemodialysis group, most of the participants were male and had an incomplete elementary school education and self-declared brown race/color. Regarding the control group, half of the participants were male, and most had completed higher education and self-declared brown race/color. 

As for biochemical tests, the hemodialysis group had a higher average of creatinine, triglycerides, and glycemia, while the control group had a higher mean value of total cholesterol. 

In addition, the variables that showed a statistically significant difference when comparing the two groups were age, schooling, tobacco use, level of physical activity, serum creatinine value, GFR value, and fasting blood glucose value. Other information can be seen in Table 1.

### 2.2. Urinary Proteomic Profile

Regarding the qualitative analyses, 416 proteins were found in the urinary proteomic profiles. Of these, 95 proteins were identified only in the control group (22.8%), 263 were detected only in the hemodialysis group (63.2%), and 58 were common to both groups (13.9%). 

**Figure 1:**
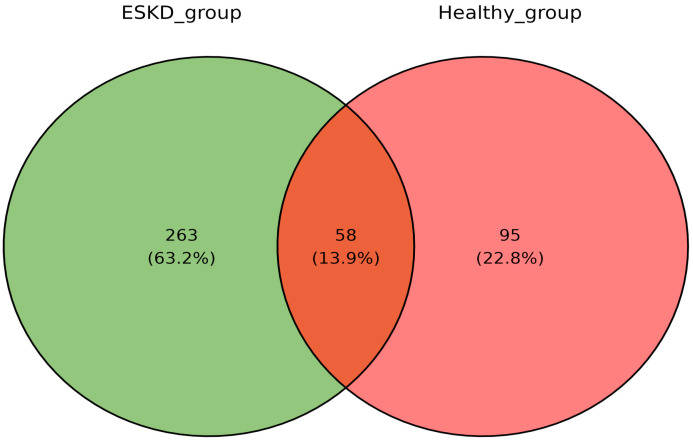
Qualitative characterization of the urinary proteomic profile of the control and hemodialysis groups. Venn diagram showing the number of proteins present in the hemodialysis group (green) and control group (red). Intersection between the circles: quantity of proteins present in both groups.

Figure 1 demonstrates the Venn diagram of the proteins found in each group and the proteins common between the groups.

**Figure 2:**
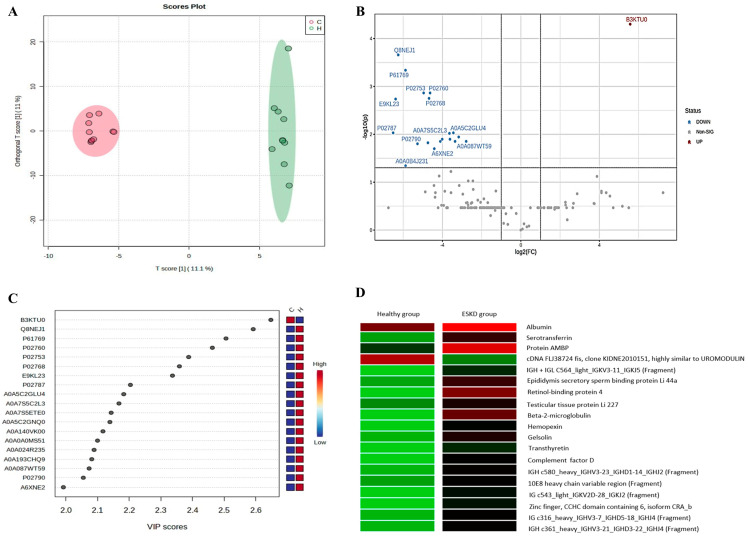
Quantitative characterization of the urinary proteomic profile of the control and hemodialysis groups. Graph (**A**)—proteins found in the control group (red) and hemodialysis group (green). Graph (**B**)—each point represents a protein; blue dots: proteins least expressed in the control group; red dot: protein most expressed in the control group; gray dots: proteins that did not show a statistically significant difference. Graph (**C**)—vertical axis: codes for the 19 proteins with a VIP score above 2.0; horizontal axis: VIP score values between 2.0 and 2.6. Graph (**D**)—green bars: absence of intensity; red bars—maximum intensity; lines: proteins; columns: healthy or hemodialysis group.

When comparing the groups quantitatively, it was seen that, of the 416 proteins initially found, 19 proteins showed a statistically significant difference. Figure 2 shows the OPLS-DA (2A) and volcano plot (2B) analyses.

In addition, Figure 2 shows the proteins that obtained VIP scores above 2.0 (2C). It is noted that 18 of the 19 statistically significant proteins were represented in the graph. The analysis of the VIP score is important because high values signal a good contribution of proteins to the separation of groups. In addition, the heatmap (2D) of the 19 significant proteins is presented, and most of these presented a higher intensity in the hemodialysis group.

### 2.3. Characterization of Significant Proteins

Supplementary Table S1 shows the significant proteins, besides the accessions collected in the UniProt database, and in which group(s) (healthy and/or ESKD group) the proteins were found.

### 2.4. Gene Ontology Analysis of Proteins

**Figure 3:**
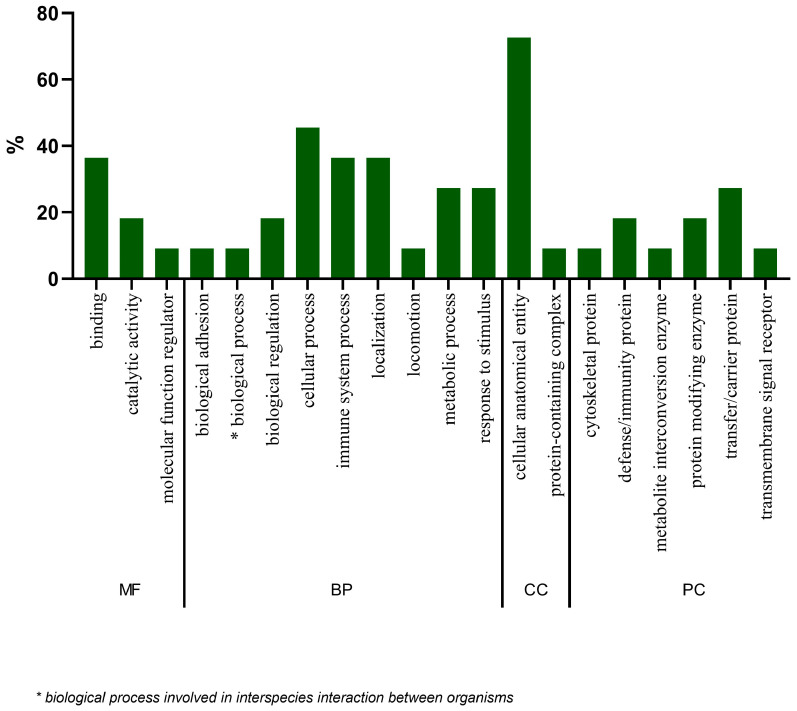
Gene ontology (GO) analysis of the 19 statistically significant proteins (*p* ≤ 0.05). MF = molecular function, BP = biological process, CC = cellular component, PC = class of proteins.

In Figure 3, the GO analysis of proteins with statistically significant differences between the two groups is presented. It is noted that in relation to molecular function and biological processes, proteins had a greater association with binding and cellular process functions, respectively. Regarding the cellular component, there is a greater participation of proteins in the cellular anatomical entity. Finally, regarding the class of proteins, it is noteworthy that these were mainly related to the class of transfer/carrier proteins.

### 2.5. Protein Interaction Analysis

**Figure 4:**
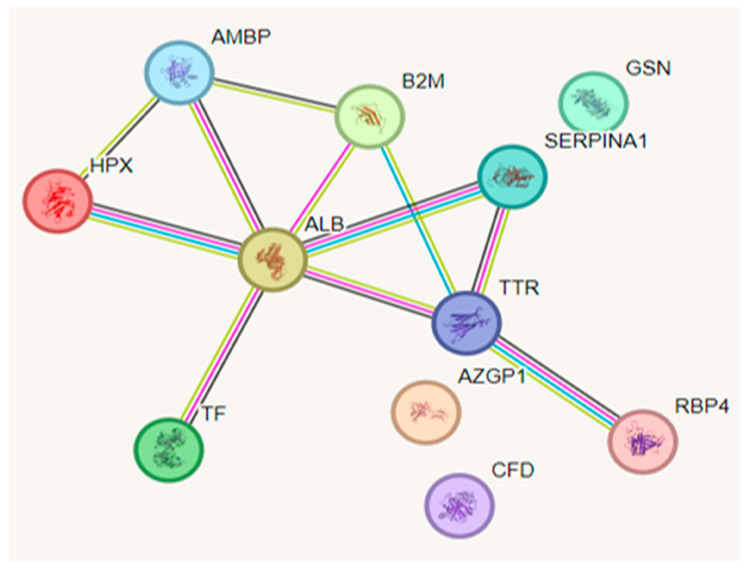
Functional interactions between proteins. B2M = beta-2-microglobulin, AMBP = alpha-1-microglobulin, ALB = albumin, HPX = hemopexin, SERPINA1 = alpha-1-antitrypsin, TTR = transthyretin, RBP4 = retinol-binding protein 4, GSN = gelsolin, AZGP1 = zinc-alpha-2-glycoprotein, TF = transferrin, CFD = complement factor D.

In Figure 4, the functional interactions found between the proteins are presented using STRING software version 12.0. It is important to highlight that the nodes are the proteins (n = 11), and the edges are the interactions between them (n = 11). Of the 19 proteins with a significant difference between the groups, eight presented interactions according to the analysis performed. 

### 2.6. Biomarker Candidate Proteins

By using the criteria for defining candidate proteins that were previously described, we found that the proteins that met all established criteria were hemopexin, beta-2-microglobulin, retinol-binding protein 4, transthyretin, and factor D. Therefore, in addition to being statistically different between the two groups, these proteins were only found in the hemodialysis group. Moreover, it was seen that there is a small elimination of beta-2-microglobulin in the urine in healthy adults. However, in cases of dysfunction of the renal proximal tubules, urinary excretion of this protein may increase [19]. In addition, hemopexin can be synthesized by the kidneys [20] and retinol-binding protein 4 can be produced in the kidneys [21], a small part of which is excreted by urine [22]. Furthermore, in cases of transthyretin mutation, it can be deposited in the kidneys and cause a condition called amyloidosis [23]. Finally, it is known that the kidneys play a role in regulating the concentration of factor D through glomerular filtration [24].

### 2.7. Accuracy Analysis of Biomarker Candidate Proteins

**Figure 5:**
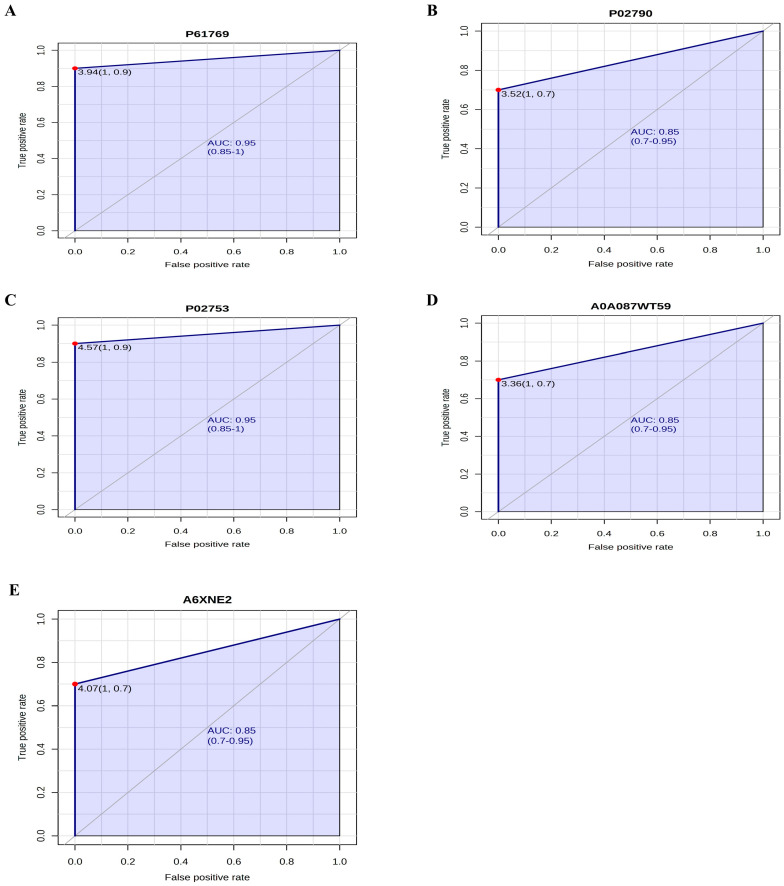
ROC curve referring to the accuracy analysis of candidate biomarker proteins (**A**) P61769; (**B**) P02790; (**C**) P02753; (**D**) A0A087WT59; (**E**) A6XNE2.

Figure 5 shows the ROC curve of the proteins beta-2-microglobulin (6A), hemopexin (6B), retinol-binding protein 4 (6C), transthyretin (6D), and factor D (6E). The proteins beta-2-microglobulin and retinol-binding protein 4 showed the same AUC value of 0.95 [with a confidence interval (CI) between 0.85 and 1]. Hemopexin, transthyretin, and factor D proteins presented an AUC of 0.85 (CI between 0.7 and 0.95). Thus, it can be affirmed that these proteins presented high accuracy for the differentiation of the groups because, according to the interpretation of the AUC, the results of the analysis of beta-2-microglobulin and retinol-binding protein 4 are considered excellent and the results of the analysis of hemopexin, transthyretin, and D factor are good.

### 2.8. Gene Ontology Analysis of Biomarker Candidate Proteins

**Figure 6:**
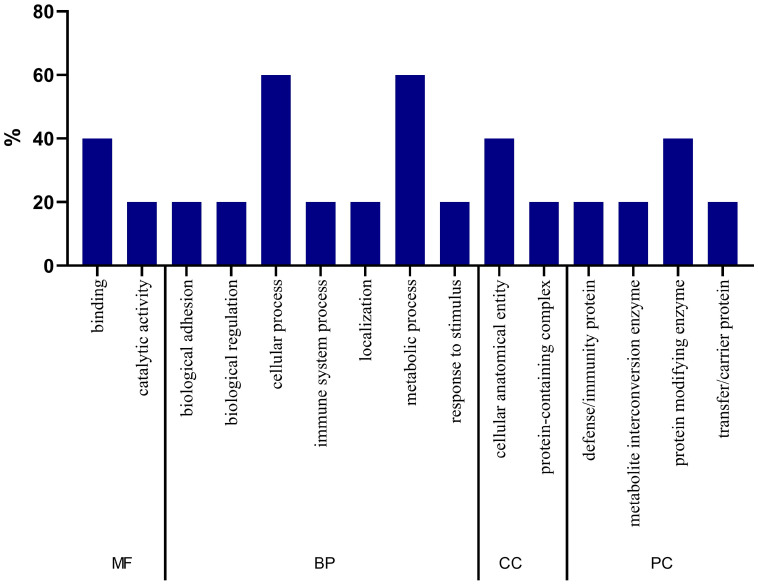
GO analysis of biomarker candidate proteins. MF = molecular function, BP = biological process, CC = cellular component, PC = class of proteins.

In Figure 6, the GO analysis of the five candidate biomarker proteins is presented. It is noteworthy that in relation to molecular function, proteins had a greater association with binding. Regarding the biological processes, these were mainly related to cellular and metabolic processes. As for the cellular component, there is a greater participation of proteins in the cellular anatomical entity. Regarding the class of proteins, these were especially related to the protein-modifying enzyme class.

## 3. Discussion

In the present study, 416 proteins were detected in the urinary proteomic profile of the control and hemodialysis groups. Of these, 19 proteins were considered statistically significant. In addition, five proteins were identified as potential candidate biomarkers for ESRD.

As for sociodemographic characteristics, the hemodialysis group had a higher mean age, which is in line with recent research showing that advanced age is associated with the onset and rapid progression of the disease [25]. It was also possible to observe that most participants in the hemodialysis group had no education or only incomplete elementary schooling. According to the National Health Survey (PNS) conducted by the Brazilian Institute of Geography and Statistics (IBGE) with a representative sample of the Brazilian population, individuals without education or with incomplete elementary schooling had a higher number of medical diagnoses of CKD when compared to people with a higher education level [26].

Regarding tobacco use, half of the participants in the hemodialysis group were former smokers. A systematic review showed that tobacco use was associated with a substantial risk of developing CKD, which can be explained by changes caused by the nephrotoxic effects of smoking, such as endothelial cell dysfunction, pro-inflammation, and oxidative stress, among others [27]. Regarding the level of physical activity of the participants, it was observed that the hemodialysis group was considered sedentary or insufficiently active. These data corroborate a study that shows that people with CKD usually have a lower level of physical activity compared to the general population and this can cause reductions in neuromuscular and cardiorespiratory activity and the worsening of quality of life. In addition, it is known that an increased level of physical activity may contribute to the deceleration of renal dysfunction [28].

Regarding the biochemical tests, as expected, the serum creatinine, GFR, and fasting blood glucose values of the participants in the hemodialysis group were higher. Increased creatinine in the blood and the consequent reduction in GFR are associated with an increased risk of CKD progression [29]. Regarding mean fasting blood glucose values, it is emphasized that persistent hyperglycemia caused by type 2 DM promotes microvascular injury and complications, such as diabetic nephropathy [30].

Concerning the proteomic analysis, the present study identified proteins that were able to characterize and differentiate the urinary proteomic profiles of the two groups. Through ontological analysis of significant proteins, it was found that there was a higher percentage of proteins that participated in biological processes that may be related to inflammation, such as cellular processes and immune system processes. Furthermore, when analyzing the ontology of candidate proteins for biomarkers, it was noted that they were associated with biological processes, such as adhesion and biological regulation, cellular and metabolic processes, immune system processes, and responses to stimulus and localization. In agreement, some studies have related these processes to low-grade systemic inflammation [31] and oxidative stress (caused by changes in metabolic processes) [32], which are present in patients with CKD and can be characterized by high levels of circulating inflammatory proteins and the presence of oxidative stress biomarkers, respectively [33]. Moreover, the accumulation of uremic toxins that occurs in CKD may contribute to the adhesion and migration of leukocytes [33,34], which increase the risk of infections and CVD in patients with CKD [34].

In addition, it was observed that the proteins that met all the established criteria to be considered biomarker candidates were hemopexin, beta-2-microglobulin, retinol-binding protein 4, transthyretin, and factor D. Through the ROC curves, it was possible to verify that these proteins showed high accuracies and were thus potential candidate biomarkers for ESRD. 

Hemopexin is an acute-phase glycoprotein, meaning that its plasma concentration may increase in the occurrence of inflammatory events [20]. The main function of this protein is to eliminate the free heme present in the systemic circulation [35]. Some studies have indicated that hemopexin is able to induce the rearrangement of the nephrine-dependent cytoskeleton in podocytes and affect the permeability of the glomerular filtration barrier by decreasing the glycocalyx, which can lead to proteinuria [35,36]. Thus, because proteinuria is an important marker of renal damage, it is suggested that increased hemopexin may be indirectly related to the occurrence of this condition and, consequently, to the development of CKD.

Beta-2-microglobulin (β2m) is a polypeptide that is bound to the main histocompatibility complex (MHC) class I protein on the surface of nucleated cells. This molecule performs the function of antigen presentation, which contributes to the proper functioning of the immune system [37]. Moreover, β2m is considered a marker of uremic toxins and is filtered exclusively by the renal glomerulus [38]. Some studies have demonstrated the association of β2m with general mortality, cardiovascular events, and disease progression in patients with CKD [38,39]. In addition, critical levels of β2m are related to a higher risk of developing dialysis-related amyloidosis [40]. Finally, it should be noted that increased levels of β2m in urine may occur in cases of dysfunction of the reabsorption of the proximal renal tubules, which can cause proteinuria [37].

Retinol-binding protein 4 (RBP4) is a serum polypeptide formed by 201 amino acids. The main sites of synthesis are the liver and adipose tissue, but it can also be produced in smaller amounts in the lungs, testes, kidneys, brain, and retina [21]. The key role of this protein is the transport of retinol from the liver to the target tissue [21]. In blood circulation, the retinol–RBP4 complex binds to transthyretin because this binding stabilizes the complex, decreases the elimination of RBP4 in renal filtration, and favors the recycling of RBP4 after the absorption of retinol in cells [21]. The retinol-free fraction of RBP4 is submitted to glomerular filtration and degraded by the proximal tubules of the kidneys [22]. However, a small portion of RBP4 is excreted in urine. Thus, when there is increased elimination of this protein in the urine, it is indicative of tubular injury [22]. In agreement, studies indicate that RBP4 can be considered a biomarker of glomerular diseases, such as diabetic nephropathy [41,42], and proximal tubulopathies, such as Falconi syndrome [42]. In addition, some studies have shown that in patients with CKD, the levels of this protein in the urine were significantly increased [22,43].

Transthyretin (TTR) is a plasma protein composed of four identical subunits containing 127 amino acids. This protein is synthesized in the liver, the choroid plexus, the pigmentary epithelia of the retina, and the ciliary of the eye and is responsible for the transport of thyroxine and the retinol–RBP4 complex [44]. In addition, when a mutation occurs in the TTR gene through the destabilization of the subunits, the protein monomers aggregate in amyloid fibrils and can be deposited in various tissues, such as the heart, nerves, kidneys, and gastrointestinal tract [23]. In the kidneys, TTR deposition may cause dysfunction of the lower urinary tract [45], nephrotic syndrome, and/or progressive renal failure [23]. Thus, in individuals with symptoms and/or a family history of amyloidosis, it is necessary to investigate the mutation through molecular tests [46]. If the mutation is proven, clinical interventions are essential to prevent the development of complications such as nephrotic syndrome and renal failure. Therefore, TTR can be considered a biomarker for the identification of CKD only in individuals with this mutation.

Factor D is a serine protease that participates in the alternative pathway of the complement system. This protein is produced and secreted into the blood circulation by adipocytes. Under healthy conditions, factor D, along with other low-molecular-weight proteins, is filtered through the renal glomerulus and completely reabsorbed inside the tubules, where it is catabolized [24]. However, in patients with ESRD, plasma levels of this protein increase by about 10-fold due to deficient glomerular filtration [24]. In addition, dysregulation of the alternative pathway may cause some inflammatory glomerular diseases, which lead to glomerular lesions and consequently hematuria and proteinuria, contributing to the development of CKD [24].

Urinary proteomics has been used in studies to identify and validate biomarkers related to different kidney diseases. A study conducted in 2024 that aimed to investigate the urinary proteome of healthy individuals and patients with CKD using the LC-MS/MS approach identified distinct protein profiles between the two groups, which also occurred in the present study. In particular, one of the proteins found (beta-2-microglobulin) was more present in the CKD group and showed a negative association with renal function, meaning that high levels of this protein were associated with a lower GFR [47]. In our study, this protein was considered a candidate biomarker for CKD.

As strengths of this study, we highlight the choice of the liquid chromatography method coupled to mass spectrometry, the quality of protein separation in the matrix by means of liquid chromatography, and the chemical identification capability of mass spectrometry equipment. Another point to be highlighted is the fact that, so far, there are few similar studies published in the literature, which ensures that this study brings additional information to this topic. In addition, the protocols used for sample preparation and analysis were validated in several studies in the scientific literature.

However, this study also has limitations: the small sample size and the absence of pools can be explained by proteomic analysis being a high-cost technique; the groups showed significant differences for tobacco consumption and physical activity, which may influence the interpretation of proteomic analysis data, as these individual factors may affect the comparison of groups; and the control group was formed by participants with a lower mean age, a fact that may also have influenced the comparison between the groups. However, this reflects the general population since advanced age is associated with higher chances of developing DM, AH, CVD, and CKD, which were exclusion criteria for the control group in the present study. Data were collected at a single time and there was no follow-up of the participants over time to verify whether the changes observed between the groups remained during this time. Finally, due to the sociodemographic and lifestyle information being self-reported, memory bias of the participants may have occurred when answering some questions. 

Regarding the limited sample size, it is worth noting that this makes it difficult to extrapolate the results found to the entire population. This can be explained by the fact that the LC-MS/MS technology used in this study has a complex methodology that requires a detailed sample preparation and processing process. However, each analysis requires high costs, which made it difficult to increase the number of participants in this study. This is a common limitation in similar studies that use LC-MS/MS technology. Nevertheless, it is essential to consider the importance of these data, since there are few studies on this topic published in the literature.

In conclusion, this study identified 416 urinary proteins, 19 of which showed important differences between healthy individuals and those with ESRD. Among these, hemopexin, beta-2-microglobulin, retinol-binding protein 4, transthyretin, and factor D stand out as potential biomarkers for ESRD due to their involvement in the pathophysiological mechanisms of the disease. These findings provide valuable information about the urinary proteomic profile of ESRD and highlight the importance of additional longitudinal studies in larger, representative populations. These efforts are essential to validate the use of these biomarkers alongside current diagnostic and monitoring methods, ultimately improving early detection and management of CKD progression.

## 4. Materials and Methods

### 4.1. Sample Design and Selection

This is a study conducted with two groups. The control group was composed of 10 healthy individuals and the hemodialysis group consisted of 10 patients with ESRD. 

The subjects of the control group were selected at the Clinical Analysis Laboratory of the Clinical Hospital of the Federal University of Uberlandia (HC-UFU). This group included those who were 18 or older and did not have diabetes mellitus (DM), arterial hypertension (AH), cardiovascular disease (CVD), or kidney disease (acute or chronic).

The subjects of the hemodialysis group were invited to the Hemodialysis Sector of HC-UFU. The inclusion criteria for this audience were aged 18 or older, presenting with an estimated GFR equal to or less than 15 mL/min/1.73 m^2^, and having undergone hemodialysis for more than one year. Patients in the HD group underwent hemodialysis via arteriovenous fistula access, three times a week, with an average time of four hours per session.

In both groups, individuals with severe clinical conditions, as well as pregnant women and individuals with a history of alcohol and/or drug abuse, were excluded.

### 4.2. Data Collection

Sociodemographic (sex, age, race/color, schooling, and family income), behavioral (smoking, alcoholism, and sedentary lifestyle), clinical (presence of comorbidities, blood pressure, and fasting glucose values), anthropometric (weight and height), and biochemistry (by collecting urine and blood samples) variables were collected. Anthropometric data and blood test results were obtained from the medical records of the participants. 

For the collection of information regarding sociodemographic, behavioral, and clinical variables, a semi-structured interview script was used. To assess the level of physical activity, the short version of the International Physical Activity Questionnaire (IPAQ) proposed by the World Health Organization (WHO) and validated in Brazil was used [48]; after the application of this questionnaire, the participants were classified as “very active”/“active” or “irregularly active”/”sedentary”. The Body Mass Index (BMI) was calculated through the relationship between weight and height squared and classified according to the criteria of Lipschitz for the elderly [49] and the WHO for adults [50]. In the case of patients in the hemodialysis group, the dry weight data were used to calculate the BMI. Adult participants (under 60 years old) were classified as eutrophic if they had a BMI of 18.5 to 24.9 kg/m^2^ and as overweight if their BMI was above 25 kg/m^2^ [50]. Elderly participants (over 60 years old) were considered eutrophic if their BMI was between 22 and 27 kg/m^2^ and overweight if their BMI was above 27 kg/m^2^ [49]. In addition, for comparison purposes, the data of “overweight” and “obesity” were categorized as “overweight”.

For the analysis of renal function, participants’ GFR was estimated using the Chronic Kidney Disease Epidemiology Collaboration (CKD-EPI) formula [2].

Urine samples from all participants were collected in sterile tubes from the brand FirstLab, after clarification on the procedure and delivery of the containers for collection. These samples were stored in the ultra-freezer at a temperature of −80 °C (Thermo Scientific: Asheville, NC, USA).

### 4.3. Ethical Aspects

This study was approved by the Ethics Committee in Research with Human Beings (CEP) of the Federal University of Uberlandia (UFU), under opinion number 4.430.315, on 1 December 2020.

According to resolution N° 466/2012 of the National Health Council, the participants signed the Informed Consent Form (ICF), after doubts were resolved and with the guarantee that they would not have their identities disclosed.

### 4.4. Proteomic Analysis

To perform the proteomic assays, pipettes of varying volumes between 0.5 and 1000 uL from the HTL brand were used. Equipment such as a centrifuge (Eppendorf 5804R: Hamburg, Germany), vortex (Kasvi K40-10208: São José dos Pinhais, Paraná, Brazil), and vacuum concentrator (MiVac Centrifugal Concentrators GeneVac™: Ipswich, Suffolk, UK) were also used.

**Figure 7:**
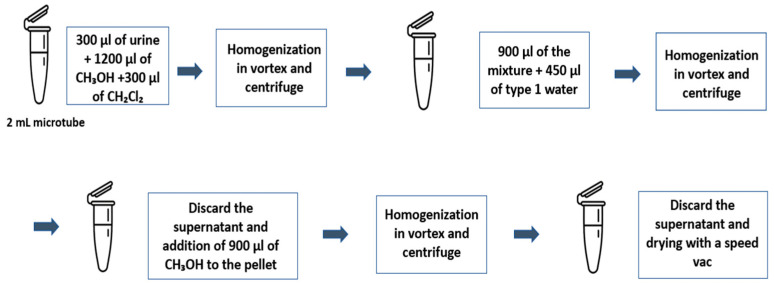
Sample preparation protocol.

Regarding sample preparation, 300 microliters (μL) of urine, 300 μL of dichloromethane (CH_2_Cl_2_), and 1200 μL of methanol (CH_3_OH) were added. After that, this mixture was homogenized by means of the vortex agitator and centrifuged at 9000× *g* for 1 min. Then, 900 μL of type 1 (ultrapure) water was added to the supernatant and the contents of the aliquots were homogenized and centrifuged at 9000× *g* for 2 min. The supernatant was discarded and 900 μL of CH_3_OH was added to the pellet, which was shaken and centrifuged. The supernatant was removed, and the pellet was dried in the vacuum concentrator. Figure 7 illustrates the sample preparation protocol.

After precipitation, the pellets contained in the microtubes were resuspended in 50 μL of type 1 water to perform colorimetric detection and protein quantification, using the Pierce TM BCA Protein Assay Kit based on bicinchoninic acid TM (BCA) according to the manufacturer’s guidelines [51].

Soon after, the digestion step was performed in solution and 75 µg of proteins in the samples were used in a final volume of 50 µL. The samples were treated with 1% surfactant (RapiGest SF—Waters: 186002123), 0.5 molar (M) dithiothreitol (DTT) (Sigma-Aldrich), and 0.5 M iodoacetamide (IAA) (Sigma-Aldrich, Darmstadt, Germany) [52]. The samples were digested by trypsin (Promega Corporation) (20 ng/μL) at 37 °C overnight.

For the desalination process of peptides, resin C18 (Omix, Agilent: Santa Clara, California, United States) was used according to the manufacturer’s guidelines. Two washes were performed in acetonitrile (ACN) 50%, followed by two washes in TFA 0.1%. The samples were passed through the column, using an up–down movement. The resin was washed again with TFA 0.1% and eluted with 100 μL ACN 50% + TFA 0.1%. Soon after, the eluted peptides were processed with a vacuum concentrator and resuspended in TFA 0.1%.

Regarding the analyses, these were performed in a liquid chromatograph (Agilent Infinity 1260) coupled to a high-resolution mass spectrometer with an electrospray ionization source (Agilent 6520B Q-TOF: Santa Clara, CA, USA). The chromatographic and ionization parameters used are described in Table 2.

The ionization parameters were optimized for the liquid chromatograph (Agilent Infinity 1260) coupled to a high-resolution mass spectrometer with electrospray ionization (ESI) source (Agilent 6520B Q-TOF). The nebulizer gas pressure was adjusted to 45 psi to ensure adequate sample atomization. The drying gas flow rate of 8 L/min and the temperature of 325 °C facilitated efficient desolvation of the droplets, optimizing ion formation. The capillary voltage was adjusted to 4 kV, providing the electric field required for efficient ionization. These conditions were standardized to maximize sensitivity and ensure analysis reproducibility.

The raw data obtained in the LC-MS/MS analysis were processed using Spectrum Mill MS Proteomics software (Agilent Technologies). This software allows the identification and quantification of peptides through the interpretation of generated mass spectra, using advanced algorithms for the analysis of proteomic data. Spectrum Mill tools include functionalities for filtering, alignment, and searching for sequences in databases, ensuring high accuracy in the identification of peptides and proteins present in the samples. The analysis of mass spectrometry (MS) data was conducted using the SpectrumMill software, incorporating specific parameters across distinct processing stages to ensure robust and reliable results. For MS extraction, spectral feature filtering was applied to precursor ions within a mass range (MH^+^) of 200 to 6000 Da. Precursor charge assignment allowed for a maximum charge state (Z) of 6, with a minimum signal-to-noise ratio (S/N) of 25 at MS1, and the detection of the ^12^C precursor *m*/*z* was enabled. In the MS/MS extraction phase, validation filters excluded spectra without validated sequences, and the batch size was set to 1000 spectra, reporting a maximum of 10 hits per batch. The analysis utilized the Uniprot database (November 2023), allowing up to five missed cleavages for peptide identification. The data acquisition was performed using an ESI Q-TOF instrument. Monoisotopic masses were considered, with precursor and product mass tolerances set at ±20 ppm. The maximum matched peak intensity was capped at 30%, and the maximum ambiguous precursor charge was limited to 6. MS/MS auto-validation was conducted using an auto-threshold strategy in peptide mode, maintaining a false discovery rate (FDR) of 1.2% across each liquid chromatography run. Peptide precursor charges were analyzed within a range of 2 to 6, and sequence lengths were constrained to a minimum of 6 and a maximum of 99 residues. Tabulated identification results were filtered to exclude unvalidated sequences. Proteins were grouped using an expanded subgrouping method, ignoring shared peptides and employing the shared group scoring (SGS) approach. Proteins were sorted by score, retaining those with a score greater than 50, while peptides were filtered for scores above 50 and with a spectrum intensity percentage (SPI) exceeding 50%. These stringent parameters collectively ensured the high confidence and accuracy of the protein and peptide identifications. In this step, *p* ≤ 0.05 was used to indicate a statistically significant difference between the groups, using the *t*-test, in addition to the FDR criterion (false discovery rate), to control false positive results [53].

### 4.5. Enrichment Analysis 

Initially, the primary and secondary accessions of the proteins considered statistically significant were collected through the Universal Protein Resource database (UniProt, https://www.uniprot.org, accessed on 5 March 2023). Then, the accessions were inserted into the Protein Analysis Through Evolutionary Relationships software (PANTHER, version 17.0, http://pantherdb.org, accessed on 6 March 2023) to perform the analysis of gene ontology (GO) proteins, which included data on molecular function (MF), biological process (BP), cellular component (CC), and protein class (PC). Finally, proteins were included in the Search Tool for the Retrieval of Interacting Genes/Proteins software (STRING, version 12.0, https://string-db.org/, accessed on 4 August 2023) to identify protein–protein interactions. The interaction network was constructed with a minimum required score of 0.900 (considered “very high”).

### 4.6. Criteria Used for the Definition of Biomarker Candidate Proteins

The criteria used to define the candidate proteins for biomarkers for ESRD were as follows: *t*-test with *p*-value ≤ 0.05 corrected by the criterion of FDR, statistically significant differential expression observed by fold-change, production and/or expression in the kidney and/or excretion in the urine, presence in only one of the groups, and a VIP score value ≥ 2.0.

### 4.7. Statistical Analysis

As for the description of sociodemographic, behavioral, and clinical variables, we used the mean and standard deviation for the quantitative variables and absolute and relative frequency for the qualitative variables. The qualitative variables were compared using the chi-square test and the quantitative variables were analyzed using the *t*-student test. Statistical data were evaluated using the Stata package (version 14.2) and the level of significance established was *p* ≤ 0.05.

Bioinformatics analyses were performed using the MetaboAnalyst Program (version 5.0). In addition, the results were expressed on the logarithmic scale of base 10 considering the spectral count. For the analysis of the proteomic profile, a Venn diagram was presented, showing the absolute number of proteins identified in each group. When comparing the two groups, orthogonal discriminant analysis by partial least squares (OPLS-DA) was presented; the volcano plot, which identifies proteins with a significant difference between the two groups (considering the *t*-test significance level of *p* ≤ 0.05 and fold-change > 2); the variable importance score in the projection (VIP score ≥ 2.0); and the heatmap, which demonstrates the average intensity of proteins with significant differences between the two groups (considering the *t*-test significance level of *p* ≤ 0.05 and fold-change > 2). Finally, an analysis of the receiver operating characteristic curve (ROC curve) of the main proteins with significant differences was performed. The area under the curve (AUC), which is considered an estimate of behavior and of the overall accuracy of the test, can be interpreted as follows: between 0.5 and 0.6 = failed; between 0.6 and 0.7 = worthless; between 0.7 and 0.8 = poor; between 0.8 and 0.9 = good; above 0.9 = excellent [54].

## Figures and Tables

**Table 1:** Characterization of the Study Participants.

Variables		Control Group (*n* = 10)	Hemodialysis Group (*n* = 10)	*p*-Value *
Sex	Male	5 (41.7)	7 (58.3)	0.361
	Female	5 (62.5)	3 (37.5)	
Age in years, mean ± standard deviation		40.3 ± 12.3	60.4 ± 8.2	<0.001
Schooling	No education/incomplete elementary schooling	0	6 (100)	0.038
	Complete high school/incomplete higher education	4 (57.1)	3 (42.8)	
	Complete higher education	6 (85.7)	1 (14.3)	
Race/color	White	4 (66.7)	2 (33.3)	0.580
	Black	1 (33.3)	2 (66.7)	
	Brown	5 (45.5)	6 (54.5)	
Marital status	With partner	6 (60.0)	4 (40.0)	0.247
	Without a partner	4 (40.0)	6 (60.0)	
Nutritional status	Eutrophic	5 (45.5)	6 (54.5)	0.337
	Overweight	5 (55.5)	4 (44.5)	
Tobacco use	Never smoked	10 (66.7)	5 (33.3)	0.010
	Ex smoker	0	5 (100)	
Alcohol consumption	Never drinks	7 (58.3)	5 (41.7)	0.607
	Less than once a month	1 (50.0)	1 (50.0)	
	Once or more a month	2 (33.3)	4 (66.7)	
Physical activity	Sedentary or irregularly active	0	7 (100)	0.024
	Active or very active	10 (76.9)	3 (23.1)	
Creatinine, mean ± standard deviation		0.9 ± 0.1	9.6 ± 1.4	<0.001
GFR, mean ± standard deviation		94.9 ± 27.1	5.4 ± 1.1	<0.001
Total cholesterol, mean ± standard deviation		186.3 ± 27.0	172.2 ± 45.1	0.713
Triglycerides, mean ± standard deviation		141.1 ± 40.2	233.1 ± 138.4	0.112
Fasting blood glucose, mean ± standard deviation		83.5 ± 4.9	124.5 ± 45.9	0.011

* Significance level *p* ≤ 0.05.

**Table 2:** Chromatographic and Ionization Parameters.

Chromatographic Parameters	Description
Column	Agilent model AdvanceBio Peptide Mapping
Internal diameter	2.1 mm
Length	10 cm
Particle size	2.7 μm
Mobile phase (A)	Water acidified with formic acid (0.1% *v*/*v*)
Mobile phase (B)	Acetonitrile acidified with formic acid (0.1% *v*/*v*)
Gradient	2% B (0 min)
	2% B (10 min)
	15% B (40 min)
	50% B (150 min)
	70% B (200 min)
	98% B (220 min)
	98% B (300 min)
	100% of B (301 min)
	100% of B (400 min)
Flow rate	400 µL/min
**Ionization parameters**	
Nebulizer pressure	45 psi
Drying gas flow rate	8 L/min
Drying gas temperature	325 °C
Capillary voltage	4 kV

Legend: millimeters (mm); centimeters (cm); micrometers (μm); per square inch (psi); kilovolts (kV).

## Data Availability

The raw data supporting the conclusions of this article will be made available by the authors upon request.

## Supplementary Materials

The following supporting information can be downloaded at: https://www.mdpi.com/article/10.3390/ijms26125429/s1. Table S1: Characterization of the 19 proteins with statistically significant differences.
