# Supplementary material for: Urine Proteomics for Detection of Potential Biomarkers for End-Stage Renal Disease
Source: Int J Mol Sci. 2025 Jun 6;26(12):5429. doi: 10.3390/ijms26125429 (PMC12193577; doi:10.3390/ijms26125429)
Supplement: Supplementary file 1 [file ijms-26-05429-s001.zip › ijms-3320363-supplementary.pdf]

Supplementary Material

Table S1. Characterization of the 19 proteins with statistically significant differences.

Protein name	Alternative names	UniProt access	Group(s) where the protein was found
Albumin	cDNA FLJ78413, highly similar to Homo sapiens albumin, mRNA;<br>cDNA FLJ95666, highly similar to Homo sapiens albumin (ALB), mRNA;<br>Serum albumin;<br>cDNA FLJ54371, highly similar to serum albumin;<br>cDNA FLJ50830, highly similar to serum albumin;<br>Isoform 2 of Albumin;<br>Isoform 3 of Albumin	P02768	Control and Hemodialysis
Serotransferrin	cDNA FLJ54111, highly similar to Serotransferrin;<br>Transferrin, isoform CRA_c;<br>cDNA FLJ56687, highly similar to Serotransferrin;<br>TF protein (fragment)	P02787	Control and Hemodialysis
Protein AMBP	Inter-alpha-trypsin inhibitor (fragment)	P02760	Control and Hemodialysis
cDNA FLJ38724 fis, clone KIDNE2010151, highly similar to UROMODULIN	Uromodulin;<br>Isoform 2 of Uromodulin;<br>Isoform 3 of Uromodulin;<br>Isoform 4 of Uromodulin;<br>Isoform 5 of Uromodulin;	P07911<br>B3KTU0	Control and Hemodialysis

	cDNA FLJ45746 fis, clone KIDNE2018727, highly similar to UROMODULIN		
IGH + IGL c564_light_IGKV3-11_IGKJ5 (Fragment)	IG c185_light_IGKV3-11_IGKJ2 (Fragment)<br>IGL c526_light_IGKV3-20_IGKJ1 (Fragment);<br>IGL c1611_light_IGKV3-11_IGKJ5 (Fragment);<br>IGL c2008_light_IGKV3-11_IGKJ4 (Fragment);<br>IGL c3277_light_ IGKV3-11_IGKJ4 (Fragment);<br>IGH + IGL c620_light_ IGKV3-11_IGKJ5 (Fragment)	A0A5C2GDK3	Hemodialysis
Epididymis secretory sperm binding protein Li 44[a]	Alpha-1-antitrypsin;<br>Epididymis secretory sperm binding protein; Isoform 2 of alpha-1-antitrypsin;<br>Alpha-1-antitrypsin (fragment);<br>PRO2275;<br>Isoform 3 of alpha-1-antitrypsin;<br>Alpha-1-antitrypsin Valcamonica variant (fragment);<br>Alpha-1-antitrypsin null variant (fragment); Serpina 1;<br>Alpha-1-antitrypsin MBrescia variant (fragment);<br>Alpha-1-antitrypsin short transcript variant 1C4;<br>Alpha-1-antitrypsin null genova variant (fragment);<br>Alpha-1-antitrypsin null (Brescia) variant (fragment)	E9KL23	Control and Hemodialysis
Retinol-binding protein 4	Retinol-binding protein	P02753	Hemodialysis
Testicular tissue protein Li 227	Zinc-alpha-2-glycoprotein;<br>AZGP1 protein (fragment)	P25311<br>A0A140VK00	Control and Hemodialysis
Beta-2-microglobulin	Beta-2-microglobulin (fragment)	P61769	Hemodialysis
Hemopexin	Epididymis secretory sperm binding protein; cDNA FLJ56652, highly similar to hemopexin	P02790	Hemodialysis
Gelsolin	Isoform 2 of gelsolin;<br>Isoform 3 of gelsolin;<br>Isoform 4 of gelsolin	P06396<br>A0A0A0MS51	Control and Hemodialysis
Transthyretin	-	P02766	Hemodialysis

		A0A087WT59	
Complement factor D	-	P00746<br>A6XNE2	Hemodialysis
IGH c580_heavy_IGHV3-23_IGHD1-14_IGHJ2 (Fragment)	IGH c2982_heavy_IGHV3-23_IGHD2-8_IGHJ2 (Fragment)	A0A7S5C2L3	Control and Hemodialysis
10E8 heavy chain variable region (Fragment)	IGH + IGL c351_heavy_IGHV3-15_IGHD5-18_IGHK4 (fragment);<br>IGH + IGL C38_heavy_IGHV3-15_IGHD1-14_IGHJ4 (fragment);<br>IG c730_heavy_IGHV3-15_IGHD7-27_IGHJ6 (fragment);<br>IG c255_heavy_IGHV3-15_IGHD3-10_IGHJ4 (fragment);<br>IG c684_heavy_IGHV3-15_IGHD3-9_IGHJ3 (fragment);<br>IG c260_heavy_IGHV3-15_IGHD3-9_IGHJ3 (fragment);<br>IG c1073_heavy_ IGHV3-15_IGHD3-22_IGHJ5 (fragment);<br>IG c155_heavy_IGHV3-15_IGHF3-22_IGHJ4 (fragment);<br>IGH + IGL c27_heavy_IGHV3-15_IGHD4-17_IGHJ4 (fragment);<br>Immunoglobulin heavy chain variable region (fragment)	A0A193CHQ9	Control and Hemodialysis
IG c543_light_IGKV2D-28_IGKJ2 (Fragment)	Immunoglobulin kappa variable 2-28; Immunoglobulin kappa variable 2-40;<br>HRV Fab N8-VL (fragment);<br>Cold agglutinin FS-1 L-chain (fragment); ACX82 (fragment)	A0A5C2GNQ0	Hemodialysis
Zinc finger, CCHC domain containing 6, isoform CRA_b	Terminal uridylyltransferase 7;<br>Isoform 4 of terminal uridylyltransferase 7; Isoform 6 of terminal uridylyltransferase 7	A0A024R235	Hemodialysis
IG c316_heavy_IGHV3-7_IGHD5-18_IGHJ4 (Fragment)	IG c573_heavy_IGHV3-7_IGHD3-3_IGHJ4 (fragment)	A0A5C2GLU4	Control and Hemodialysis
IGH c361_heavy_IGHV3-21_IGHD3-22_IGHJ4 (Fragment)	IGH + IGL c36_heavy_IGHV3-15_IGHD3-3_IGHJ6 (fragment);<br>IG c775_heavy_IGHV3-15_IGHD3-10_IGHJ4 (fragment)	A0A7S5ETE0	Control and Hemodialysis
